# New gene models and alternative splicing in the maize pathogen *Colletotrichum graminicola* revealed by RNA-Seq analysis

**DOI:** 10.1186/1471-2164-15-842

**Published:** 2014-10-02

**Authors:** Ivo Schliebner, Rayko Becher, Marcus Hempel, Holger B Deising, Ralf Horbach

**Affiliations:** Interdisciplinary Center for Crop Plant Research, Martin-Luther-University Halle-Wittenberg, Betty-Heimann-Str. 3, D-06120 Halle (Saale), Germany; Institute for Agricultural and Nutritional Sciences, Martin-Luther-University Halle-Wittenberg, Betty-Heimann-Str. 3, D-06120 Halle (Saale), Germany

**Keywords:** *Colletotrichum graminicola*, Anthracnose of corn, RNA-Seq, Genome annotation

## Abstract

**Background:**

An annotated genomic sequence of the corn anthracnose fungus *Colletotrichum graminicola* has been published previously, but correct identification of gene models by means of automated gene annotation remains a challenge. RNA-Seq offers the potential for substantially improved gene annotations and for the identification of posttranscriptional RNA modifications, such as alternative splicing and RNA editing.

**Results:**

Based on the nucleotide sequence information of transcripts, we identified 819 novel transcriptionally active regions (nTARs) and revised 906 incorrectly predicted gene models, including revisions of exon-intron structure, gene orientation and sequencing errors. Among the nTARs, 146 share significant similarity with proteins that have been identified in other species suggesting that they are hitherto unidentified genes in *C. graminicola*. Moreover, 5′- and 3′-UTR sequences of 4378 genes have been retrieved and alternatively spliced variants of 69 genes have been identified. Comparative analysis of RNA-Seq data and the genome sequence did not provide evidence for RNA editing in *C. graminicola*.

**Conclusions:**

We successfully employed deep sequencing RNA-Seq data in combination with an elaborate bioinformatics strategy in order to identify novel genes, incorrect gene models and mechanisms of transcript processing in the corn anthracnose fungus *C. graminicola*. Sequence data of the revised genome annotation including several hundreds of novel transcripts, improved gene models and candidate genes for alternative splicing have been made accessible in a comprehensive database. Our results significantly contribute to both routine laboratory experiments and large-scale genomics or transcriptomic studies in *C. graminicola*.

**Electronic supplementary material:**

The online version of this article (doi:10.1186/1471-2164-15-842) contains supplementary material, which is available to authorized users.

## Background

*Colletotrichum graminicola* is a filamentous ascomycete that causes anthracnose leaf blight and stalk rot of maize leading to estimated annual yield losses in the US of about 6%. In addition to its economical importance, *C. graminicola* is among the best characterized and most tractable fungi of the genus *Colletotrichum*, a genus comprising a broad range of hemibiotrophic plant pathogens that represent a constant threat to fruit and vegetable production worldwide (http://www.broadinstitute.org).

Interesting aspects of *Colletotrichum* pathology are the pressure-driven penetration strategy and the hemibiotrophic lifestyle that is featured by a fundamental switch in nutrition. An initial short biotrophic phase, during which the host cell remains alive, is followed by highly destructive necrotrophic development characterized by extended areas of killed host tissue [1].

Following germination of conidia, specialized infection cells called appressoria are differentiated on the host epidermis. Accumulation of osmolytes in appressoria of *C. graminicola* generates a hydrostatic pressure corresponding to approx. 5.5 MPa, which is among the highest turgor pressures known in living cells [2, 3]. This enormous pressure is utilized by the fungus to forcefully penetrate plant epidermis cells [4]. Upon successful penetration of the host epidermis, biotrophic hyphae of *C. graminicola* initially colonize living plant cells as evidenced by the integrity of the host plasma membrane [5, 6]. This stage of symptomless parasitic development is terminated at the onset of necrotrophy. Fast-growing hyphae breach the walls of adjacent cells, ramify within the leave and kill host cells, leading to massive destruction of maize tissue and visible necrosis. Finally, acervuli are formed on necrotic tissue harboring millions of conidia that serve as secondary inoculum. These conidia are distributed by splashing and wind-dispersed raindrops which ensures efficient dispersal and hence spread of the disease [7].

Measures to combat fungal infections require detailed knowledge of the fungal infection biology. Although some aspects of the pathogenic development of *C. graminicola* are well-understood, the molecular basis underlying the fundamental morphological and physiological switches that are indispensable for virulence of *C. graminicola* is still elusive. Large-scale genomics and transcriptomics approaches have been proven to enable the identification of pathogenicity genes in fungi affecting humans or plants [8], which makes them valuable tools towards a better understanding of fungal infection mechanisms.

A first step towards the application of such methods in *C. graminicola* research was the release of the annotated genome assembly in 2009 by the *Colletotrichum* Sequencing Project group (http://www.broadinstitute.org). The genome of *C. graminicola* was sequenced with a coverage of about 9-fold using a combination of 454 and Sanger Whole Genome Shotgun methodology. Paired-end reads from 468,734 plasmids and 67,151 fosmids were employed to improve the continuity of the assembly. Protein-coding genes were annotated using multiple lines of evidence from BLAST, PFAM searches and EST alignments, and gene structures were predicted using the Broad Institute automated gene-calling pipeline as described [9]. Further analysis revealed an exceptionally large and diverse inventory of cell wall-degrading enzymes and secondary metabolism enzymes. Moreover, 177 genus-specific genes were identified that potentially encode effector proteins. Based on data obtained from optical mapping, 14.6% of the genome could not be matched to chromosomes due to extensive repetitive sequences that were estimated to comprise 22.3% of the *C. graminicola* genome. The size of the assembled genome of *C. graminicola* was found to be 50.9 Mb with a total of 12,006 genes, 295 tRNAs and 60 rRNAs. The identity of 2,766 genes has been confirmed by sequencing of 28,424 ESTs; however, the majority of gene models still lack experimental support [9].

Predictions of gene models that are predominantly based on automated algorithms have been shown to be often incorrect or partially correct, especially for species-specific and non-conserved genes [10, 11]. Accordingly, using traditional bioinformatics tools makes it difficult to identify novel genes, UTRs, intron boundaries and alternative splicing. Since the quality of further comparative and functional genomics or transcriptomics studies depends largely on the correctness of predicted gene models, large-scale transcript information is required to improve the current annotation of the *C. graminicola* genome.

In this context, next generation sequencing technology has become a powerful tool with the potential to provide a global view not only of the genes present in *C. graminicola*, but also of their transcriptional and post-transcriptional regulation at single-nucleotide resolution. In fact, application of transcript data derived from RNA-Seq approaches offers the opportunity to identify sophisticated mechanisms of gene regulation, e. g. alternative splicing, RNA editing or the presence of regulatory RNAs [12, 13].

Recent advances in gene model prediction based on the application of RNA-Seq have been published in a number of research articles. For example, in *Sordaria macrospora* both UTR regions of about 5000 of the predicted genes could be identified, in addition to approx. 1000 gene models that were improved or newly annotated [14]. Using RNA-Seq data, Zhao and coworkers [15] identified 2459 novel transcriptionally active regions and revised 655 incorrectly predicted gene models in *Fusarium graminearum*. Moreover, 231 genes were identified with two or more alternative splice variants. A similar approach by Wang et al. [16] yielded approx. 700 novel protein-coding genes, 800 new candidate exons, UTR regions of more than 4000 genes and as many as 1032 genes differentially spliced in *Aspergillus oryzae*. These numbers impressively show the additional benefit provided by a transcript-based revision of the genome annotation.

In order to improve the predicted gene models in *C. graminicola*, we used an extensive set of RNA-Seq data covering the transcriptome of this fungus during pathogenic development in maize leaves. In this study, we revised predicted gene models, identified novel genes and nTARs, and searched for alternative splicing and RNA editing in *C. graminicola*. Based on the analysis of our RNA-Seq data, an improved annotation of the *C. graminicola* genome together with a comprehensive overview of alternatively spliced genes has been made available (http://www.landw.uni-halle.de/download/) in order to promote *Colletotrichum* research.

## Results

### Quality analysis of RNA-Seq data

We totally obtained 1.31 billion reads of a length of 100 nucleotides. Of these, approx. 51 million quality-processed reads with an average length of 82 nucleotides mapped to the genome of *C. graminicola*. This significant difference can be explained by the relatively low amount of fungal biomass that is present in the host, especially during the early stages of infection. Since about 67% of the *C. graminicola* genome contains protein-coding sequence information, the average coverage of these regions was approx. 122-fold.

**Figure 1:**
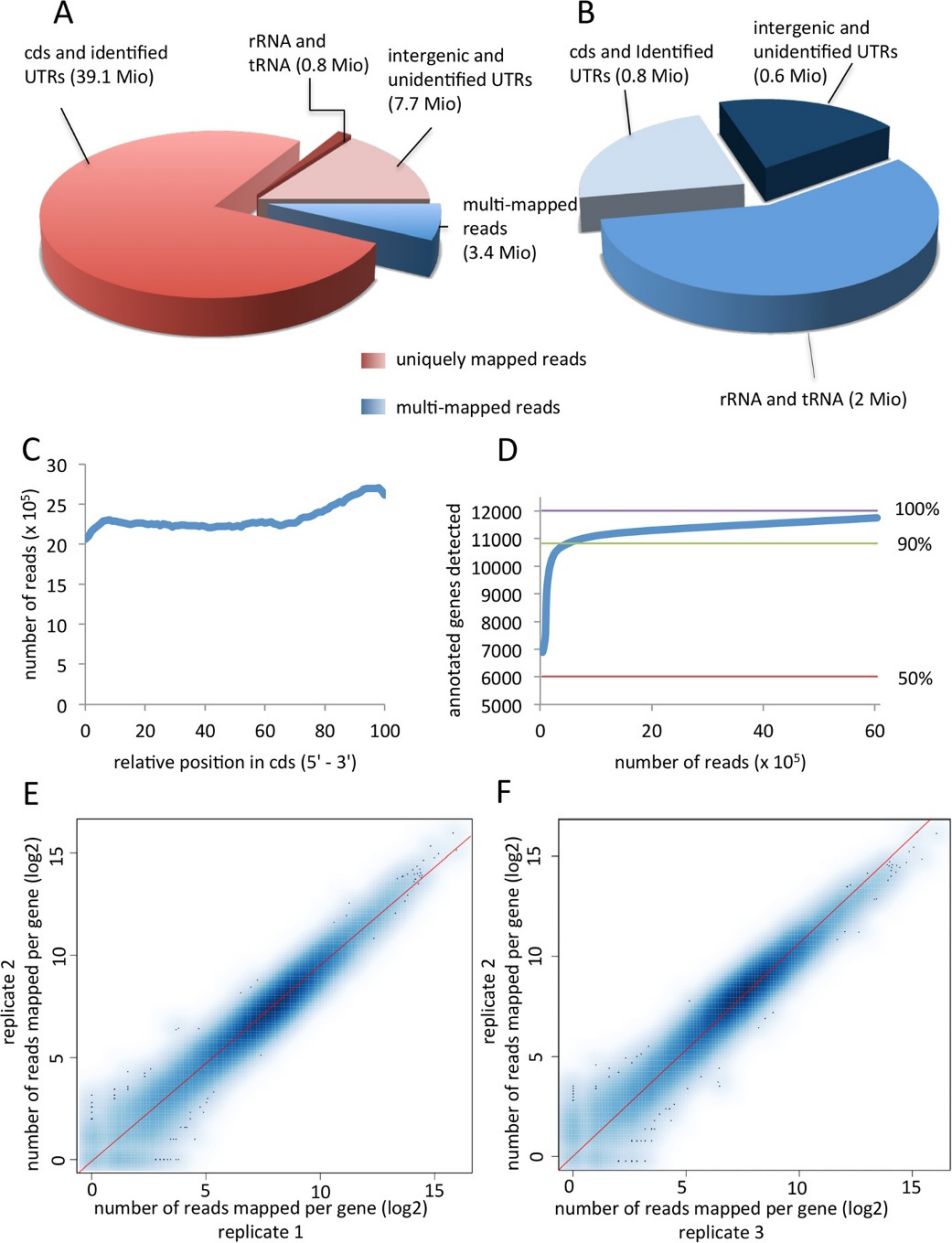
**Statistics and analysis of the quality of RNA-Seq data. A**. Distribution of mapped reads over exons, introns, intergenic and untranslated regions (UTRs). About 93% of all reads mapped to unique locations in the genome of *C. graminicola*, of which 82.5% matched coding sequences (cds) and identified UTRs, 15.9% to intergenic sequences and unidentified UTRs, and 1.6% to sequences encoding rRNA/tRNA. **B**. The remaining 7% of reads mapped to multiple locations, of which 56.8% matched sequences encoding rRNA/tRNA, 23.3% to cds and identified UTRs, and 19.9% to intergenic sequences and unidentified UTRs. The read numbers of each category are given in brackets. **C**. Total coverage of cds by RNA-Seq reads. The cds were divided into 100 equal windows. **D**. Gene coverage vs. sequencing depth. Approx. 87% of *C. graminicola* genes were detected at about 2.5 million uniquely mapped RNA-Seq reads, and coverage reaches a plateau afterwards despite the increasing sequencing depth. **E-F**. Scatter plot analysis of RNA-Seq data of biological repeats 1 vs. 2 **(E)** and 2 vs. 3 **(F)**. Each repeat consists of a combined data set of six individual RNA samples taken at 0, 12, 24, 48, 72, 120 hours postinoculation. Each blue point represents a particular gene. The color shading correlates with the density of these points.

Analysis of the localization and distribution of the obtained reads in the genomic context revealed that among the matched reads 93% were uniquely mapped to the genome with their full read length allowing two mismatches. About 7% mapped to multiple locations in the genome, of which 96% matched 2 to 10 different locations, and 4% to more than 10 different locations. RNA-Seq data showed that 82.5% of the reads that matched unique regions could be mapped to known coding/UTR regions, 15.9% to intergenic/unknown UTR sequences, and 1.6% mapped to genomic regions encoding rRNA and tRNA (Figure 1A). Of all reads that mapped to multiple locations, 56.8% mapped to genomic regions encoding rRNA and tRNA, 23.3% mapped to known UTR/protein coding regions, and 19.9% mapped to intergenic regions and yet unidentified UTRs (Figure 1B).

An indispensable prerequisite for reliable RNA-Seq data is the sufficient coverage of transcripts, preferably by a large number of reads that are evenly distributed across the target sequences [17]. Although 3-prime ends of transcripts were found to be represented by a somewhat higher quantity of reads, the overall coverage shows a largely even distribution and sufficient coverage of the coding transcript sequences by RNA-Seq reads (Figure 1C).

Altogether, transcripts corresponding to 11,732 (97.7%) of the 12,006 genes predicted in the current annotation of the *C. graminicola* genome could be detected. This number contrasts the 2,766 genes that had been previously validated by EST contigs [9]. About 9,675 (80%) of all predicted genes were found to be covered in full-length by RNA-Seq reads. Reads corresponding to about 87% of *C. graminicola* genes were detected at approx. 2.5 million uniquely mapped RNA-Seq reads. Subsequently, gene coverage increased only moderately despite the increasing sequencing depth (Figure 1D).

Further quality evaluation was performed in order to assess the reproducibility of biological repeats. Merged data sets of biological repeats 1 vs. 2 and 2 vs. 3 show consistent distribution of data points in the scatter plots (Figure 1E, 1F).

These results clearly demonstrate the good quality of the obtained RNA-Seq data as proven by the extensive coverage of hitherto identified genes, sufficient depth of the sequencing approach and the broad diversity of RNAs transcribed during pathogenic development of *C. graminicola*, which is a prerequisite for both the identification of a large number of nTARs and improvement of the annotated gene models.

### Identification of novel gene models and non-coding RNAs

In *C. graminicola*, a total of 12,006 genes, encoding proteins of more than 100 amino acids, have already been predicted by gene-finding software tools [9]. However, 15.9% of the mapped reads fell within intergenic/unidentified UTR regions. A substantial part of these reads correspond presumably to non-coding RNAs and transcripts of as yet unrecognized genes or exons of incorrectly annotated genes.

**Figure 2:**
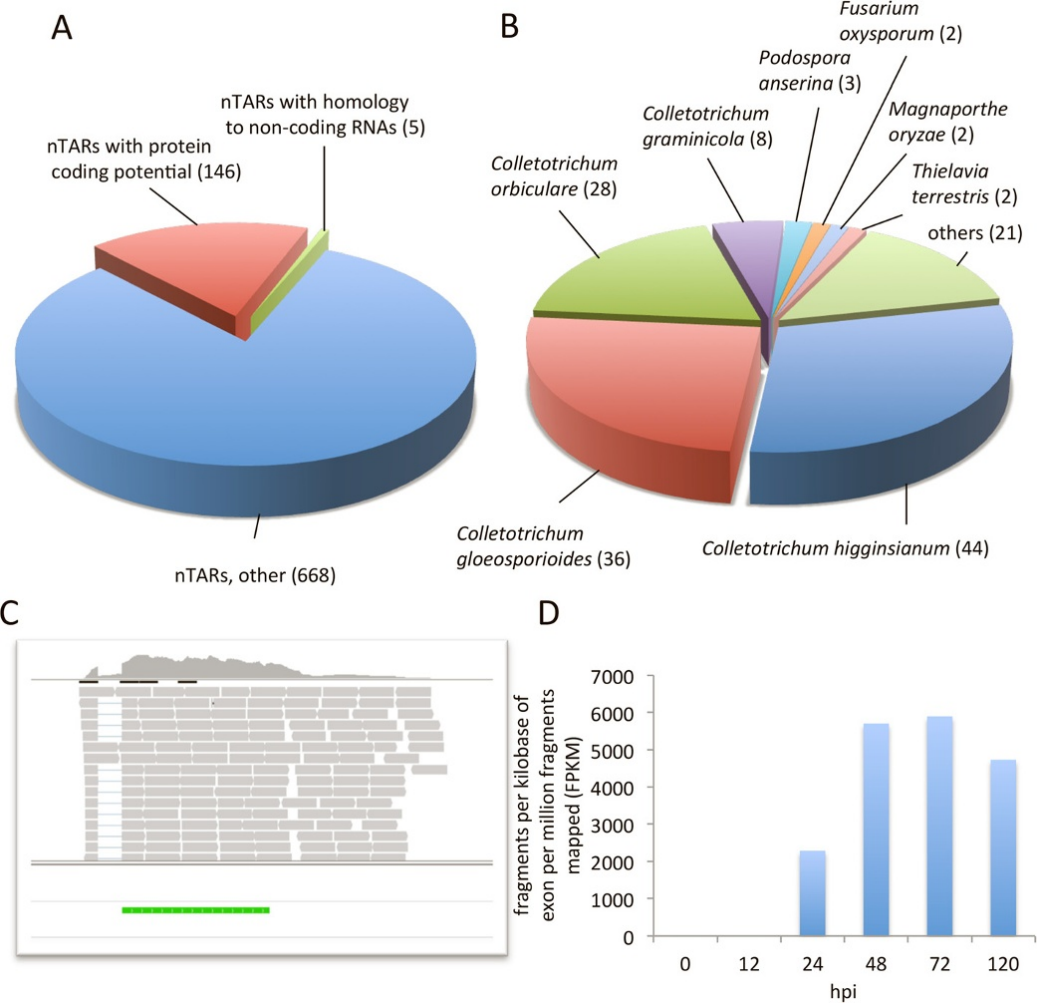
**Identification of novel gene models and non-coding RNAs. A**. Of the 819 novel transcriptionally active regions (nTARs) identified, 5 nTARs display similarity with non-coding RNAs and 146 with known proteins (E-value 10^-8^). **B**. About 80% of these share significant identity with proteins from other *Colletotrichum* species. **C**. One example of a novel transcript that has not been identified by automated annotation of the genomic sequence. Blast analysis revealed a protein of 127 amino acids with similarity (E-value 10^-34^) to the putative effector ChEC22 from *Colletotrichum higginsianum*. The green bar highlights the cds of the ChEC22 ortholog, grey bars represent individual RNA-Seq reads. **D**. Transcript profile of the Chec22 ortholog in *C. graminicola*. Transcriptional activity at 24-120 hours postinoculation (hpi) correlates with necrotrophic development of *C. graminicola* in maize leaves.

Altogether, we found 819 nTARs, of which 430 harbor introns indicating that they could be genes. In addition, about 60% of all nTARs were longer than 500 bp (Additional file 1). Conceptional 6-frame translations employed to search the non-redundant protein database of NCBI (ftp://ftp.ncbi.nlm.nih.gov/blast/db/) and the protein domain database of the Sanger-Institute (ftp://ftp.sanger.ac.uk/pub/databases/Pfam) revealed the presence of coding sequences with similarity to known proteins in 146 nTARs (E-value <10^-8^), of which 31 displayed a conserved protein domain (E-value <10^-3^, Figure 2A, Additional file 2). Notably, 74% of these putative proteins were found to have orthologs in other *Colletotrichum* species (Figure 2B).

The majority of nTARs could not be assigned to particular genes, probably due to the fact that they either lack conserved orthologous genes in other species or these genes have not been annotated so far. Alternatively, these TARs could correspond to non-coding or regulatory RNAs. In order to identify non-coding/regulatory RNAs we compared database entries of known non-coding RNAs with nucleotide sequences of the *C. graminicola* nTARs. Despite the enrichment of mRNA sequences carrying terminal poly(A)-tails during sample preparation we found 5 nTARs with similarity to non-coding RNAs, i. e. 2 transfer RNAs, 1 spliceosomal RNA,1 small nuclear RNA and 1 ribosomal RNA (Figure 2A, Additional file 3). None of the nTARs shared similarity with database entries of known regulatory RNAs. One reason for this could be that regulatory RNAs are species-specific, which would prevent identification of similar RNAs in public databases containing predominantly entries of mammals and *Drosophila melanogaster*.

Though the function of the majority of nTARs remains elusive, 106 of the nTARs that were found to have orthologs in other fungal species display intact full-length ORFs (Additional file 2). These results convincingly show that a substantial proportion of nTARs are most likely transcripts of protein-encoding genes rather than pseudogenes or regulatory RNAs. As a consequence, a significant number of genes, some of which with putative functions in virulence or pathogenicity, have not been recorded in the current version of the genome annotation. For example, an nTAR designated COGR_14733.1 (Figure 2C) encodes a protein with assumed effector function as evidenced by the occurrence of an ortholog with putative effector function in *C. higginsianum* [18]. Transcripts of COGR_14733.1 were detected exclusively between 24-120 hpi (Figure 2D), which could be indicative of a role in the switch from the biotrophic to the necrotrophic lifestyle.

### Identification of incorrect gene models

Following mapping and assembly of the RNA-Seq reads, we conducted a thorough manual inspection of the currently annotated genes using the integrative genome viewer [19] that allowed simultaneous survey of mapped reads, genome annotation of the Broad Institute and a genome annotation based on the algorithms of AUGUSTUS, a gene prediction software dedicated to the analysis of fungal genomes that has been trained using the supplied *Fusarium graminearum* data set [20]. Based on the coverage of the genomic sequence by RNA-Seq reads the following categories of incorrectly annotated features have been identified:

**Figure 3:**
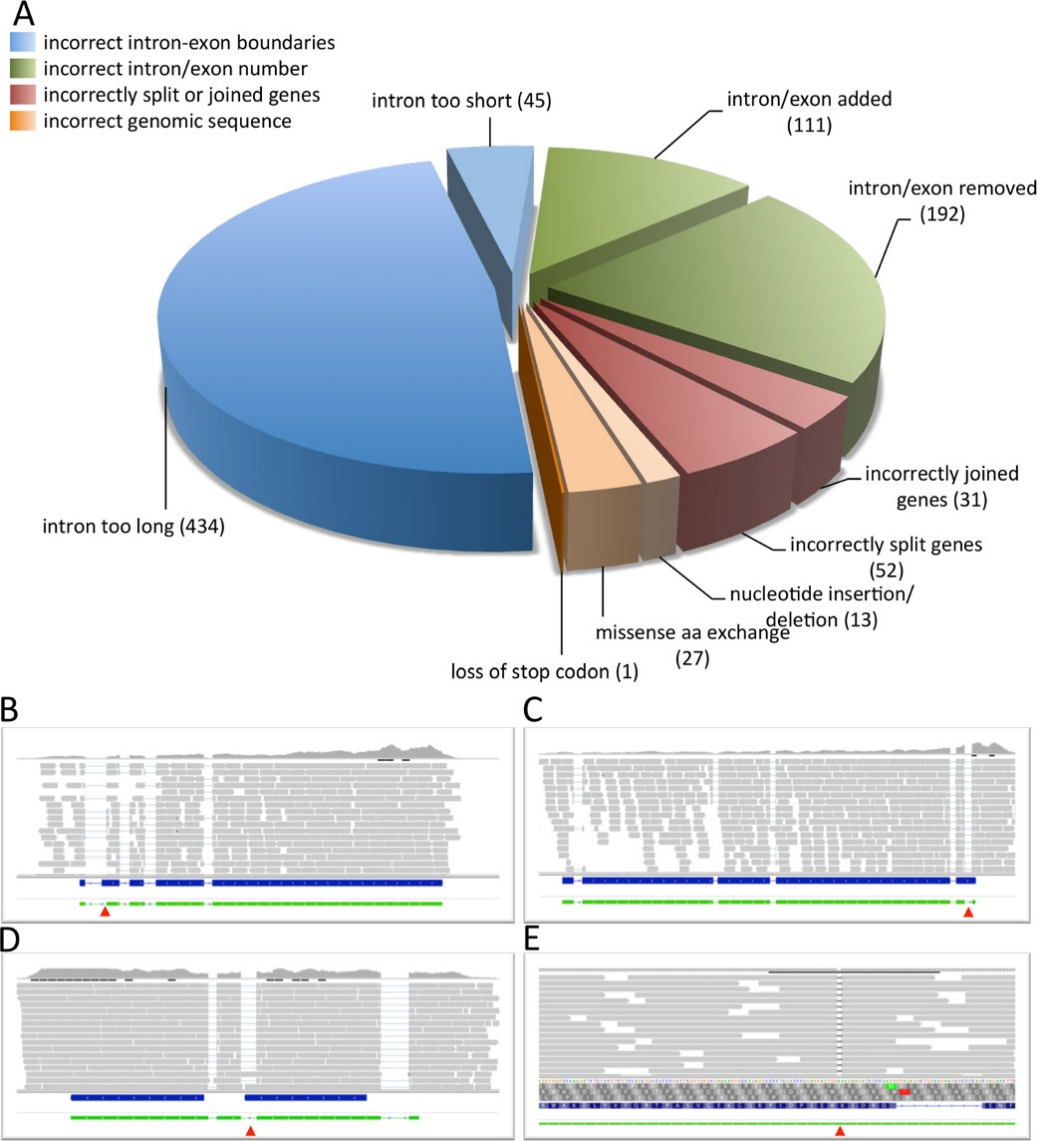
**Identification of incorrect gene models in the** ***C. graminicola*** **database. A**. Statistical analysis of incorrect gene models. In total, 906 gene models were improved with respect to incorrect introns or exons, incorrect splice sites, incorrectly split or joined genes and sequencing errors. **B-E**. Examples of the four categories of incorrectly annotated genes. **B**. Read coverage of the coding sequence of GLRG_02449 convincingly shows that the first intron is somewhat longer than predicted. C. Exon five of GLRG_06933 harbours an unidentified intron. Using RNA-Seq data, an additional short exon at the immediate 3′-terminus needed to be added to the gene structure. **D**. Gene GLRG_08413 and GLRG_08414 were incorrectly split due to the occurrence of two introns (blue bars). The correct annotation shows a single ORF that consists of four exons (green bar). **E**. Insertion of an adenine into the genomic DNA sequence of GLRG_08997 led to frame-shift and premature stop of translation. In the current annotation, the conflict was apparently solved by inserting an intron which could be identified as incorrect. Blue bars indicate incorrect cds predicted by automated gene annotation, green bars highlight the re-annotated gene structure based on RNA-Seq data, grey bars represent individual RNA-Seq reads and red arrowheads indicate the positions of errors in the current annotation of the *C. graminicola* genome.

I. Incorrect intron-exon boundaries: This type of incorrect annotation was identified either by reads that mapped partially to annotated intron regions or partial missing coverage of annotated exon regions. Prerequisite for the correction of these errors was a sufficient read coverage of the genes to be re-annotated. In comparison with the current annotation we found 434 introns that were too long and 45 that were too short (Figure  3A,B; Additional file 4).
II. Incorrect exons/introns: The procedure that led to the identification of this type of error was similar to the approach chosen to identify errors of type I with the exception that only absent or redundant full-length introns were captured. At least one intron and one exon needed to be added to the sequence of 111 genes whereas one intron and one exon was removed in 192 genes (Figure 3A,C; Additional file 4).
III. Incorrectly split or joined genes: This category refers to adjacent genes that were incorrectly joined in the annotation of the Broad Institute or individual genes that were incorrectly split due to misinterpretation of the genomic sequence data. Incorrectly joined genes were identified by the lack of read coverage in the sequence of the putative gene which could not be explained by the occurrence of an intron. Vice versa, incorrectly split genes could be found by a largely uniform read coverage throughout the sequence of the incorrectly annotated gene. In both cases, newly annotated genes were subjected to Blast search in order to confirm the identity of the new gene by sequence comparisons with orthologous genes of closely-related fungal species. In total, 31 incorrectly joined genes and 52 incorrectly split genes could be identified (Figure 3A, 3D; Additional file 4).
IV. Incorrect genomic sequence: By comparing the genomic nucleotide sequence provided by the Broad Institute with our RNA-Seq and genomic sequence data we were able to detect sequencing errors or point mutations that led to incorrect gene models (Figure 3A). Among the 81 aberrations identified, 40 did not alter the genetic information of coding sequences, i. e. they were silent nucleotide substitutions, insertions or deletions within intergenic regions and nucleotide substitutions located either upstream or downstream of coding sequences or within TARs (Additional file 5). The gene structure or sequence of the translation product was affected in 41 genes by nucleotide substitutions leading to missense, loss of the stop codon and insertions or deletions of nucleotides in coding sequences (Additional file 4).

In some cases, these errors had fatal effects with respect to the quality of the algorithm-based gene annotation. For example, an insertion of adenine at nucleotide position 948 of gene GLRG_08997 encoding a cyclohexanone monooxygenase resulted in a frameshift and hence stop of translation. Automated annotation by the Broad Institute led to the incorrect insertion of a short intron in order to solve the conflict. The correct ORF was restored by removing the adenine (Figure 3E) as proven by blastp homology search against the NCBI database. Altogether, incorrect annotations of 41 genes originating from substitutions, deletions or insertions of single nucleotides could be improved by manual revision of gene models.

Using RNA-Seq data, a total of 906 incorrect gene models identified in the current genome annotation were successfully revised, which will significantly contribute to both increased quality and reliability of the genomic data of *C. graminicola*.

### Identification of alternative splicing (AS)

**Figure 4:**
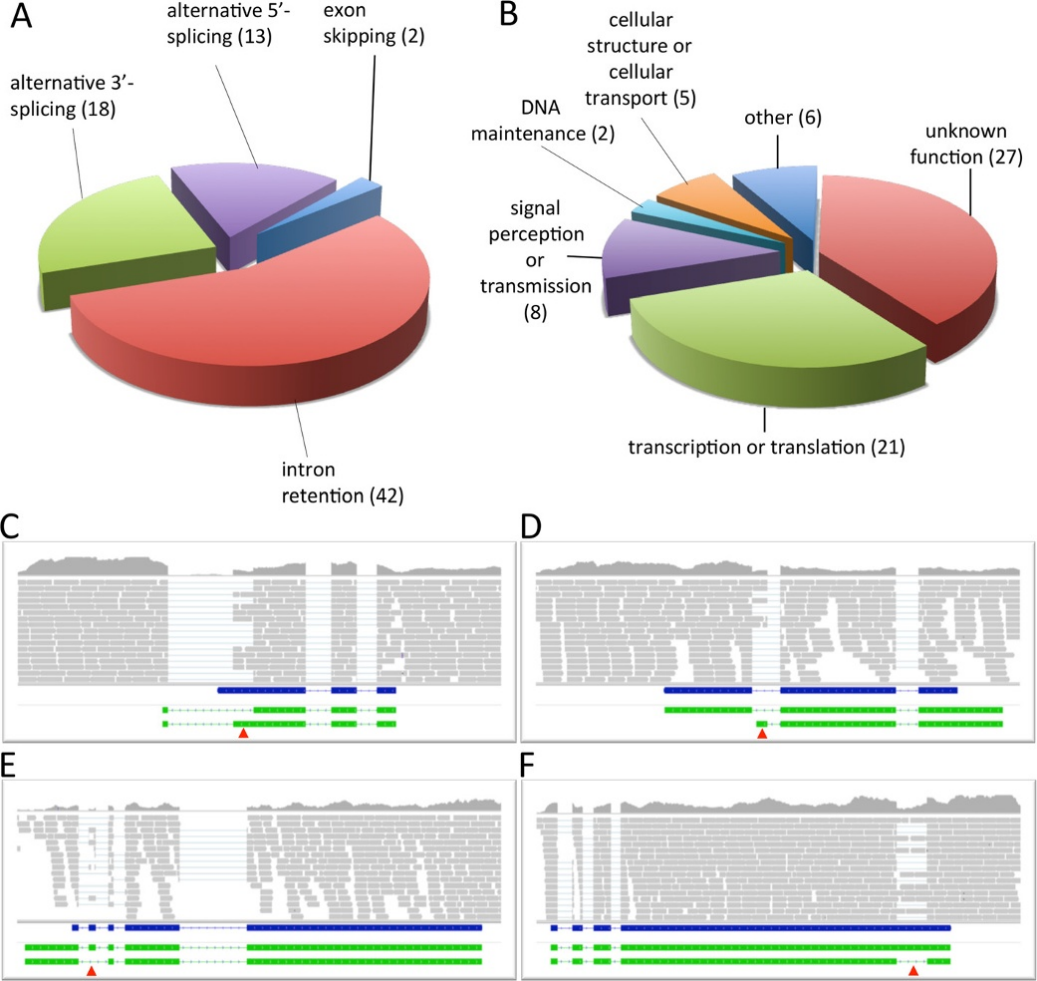
**Identification of alternative splicing. A**. Overview of types and frequency of putative alternative splicing events in *C. graminicola*. **B**. Classification of alternatively spliced genes according to their functions. **C**. Alternative 3′- or **(D)** 5′- splice sites were identified by a significant number of reads covering intron sequences at their 3′- or 5′-ends whereas intron retention **(E)** could be identified by reads covering the entire intron sequence. Exon skipping **(F)** was identified by a significant number of reads that cover predicted intron sequences, but do not bridge the gap between the alternate exon and adjacent exons. Blue bars visualize the structure of gene models predicted in the current annotation of the *C. graminicola* genome. Manually improved alternate gene model are illustrated by green bars, grey bars represent individual RNA-Seq reads.

Alternative splicing describes the regulated processing of mRNAs that leads to the expression of multiple proteins encoded by a single gene, which is an inevitable prerequisite for diversity and functional complexity of eukaryotic proteomes [21–23]. In order to assess alternative splicing in *C. graminicola* we inspected the re-annotated genome with special emphasis on reads that match intronic regions. Candidate genes for alternative splicing were identified by a significant read coverage within intron sequences as compared to the read coverage of the surrounding exons. We found 75 putative AS events in 69 genes, including exon skipping, intron retention and alternative 5′ or 3′ splice sites (Figure 4A, Additional file 6).

Intron retention appears to be the most prevalent form of AS in *C. graminicola*, accounting for about 56% of all AS events. However, analysis of the alternate ORFs revealed premature termination of translation in most of the candidate genes irrespective of the AS mechanism. There was no evidence for a stage-specific exchange of entire domains, a characteristic effect of AS in higher organisms that leads to functionally diverse proteins.

The majority of the AS candidate genes that have been characterized are involved in transcription or translation. Further putative functions include maintenance of DNA, signal perception or transmission and cellular structure or transport (Figure 4B).

In total, about 0.6% of all *C. graminicola* genes were estimated to undergo AS, though it must be emphasized that delayed processing of premature mRNAs may mimic alternative splicing [24, 25]. In line with this, detection of alternatively spliced RNAs by RT-PCR, which has been frequently used, does not appear to be an appropriate technique providing sufficient evidence for alternative splicing.

### Identification of untranslated regions (UTRs)

We identified the 5′- and 3′-boundaries of transcripts by searching for a sharp decline of RNA-Seq reads signals at both ends of the annotated transcripts. UTRs of genes that overlap with UTRs of adjacent genes were excluded from further analysis. Altogether, 5′- and 3′-UTRs of 4378 genes could be retrieved (Additional file 7). The average length of UTRs in the genome of *C. graminicola* was found to be 272 nucleotides. UTRs of the 5′- and 3′-regions have an average length of 229 and 316 nucleotides, respectively. Among the identified UTRs, 893 harbor intronic sequences, of which 535 are located in the 5′-UTRs and 363 in the 3′-UTRs.

### Screening for RNA editing

Comparing the genomic sequence and RNA-Seq data obtained from *C. graminicola* strain M2 offered the opportunity to identify nucleotide modifications that occurred due to RNA editing.

A genome-wide search for SNPs, deletions and insertions (INDELS) of single nucleotides yielded 81 of these modifications, however, careful manual examination revealed that the majority of putative RNA editing-based substitutions or insertions were restricted to repetitive sequences of a particular nucleotide or located near intron splice sites. Therefore, we concluded that single nucleotides were erroneously introduced or deleted by DNA polymerases used in the sequencing reactions rather than by RNA editing mechanisms. In addition, several SNPs appeared to be caused by misalignment of RNA-Seq reads to the genomic DNA sequence. In order to verify the nucleotide sequence of regions harboring putative SNPs we performed local re-sequencing of the genomic DNA. The obtained results strongly suggest that no RNA editing occurs in *C. graminicola*.

## Discussion

In the present study we employed an extensive set of RNA-Seq data covering the infection-related transcriptome of the maize pathogen *C. graminicola* to evaluate the correctness and completeness of the predicted gene models that are present in the annotated genome database hosted by the Broad Institute. Moreover, detailed sequence information of transcripts obtained by RNA-Seq enabled the investigation of post-transcriptional processing and regulation in *C. graminicola,* such as RNA editing or alternative splicing.

Deep sequencing techniques provide considerable advantage over microarrays that have been frequently used in transcriptome analysis [26–28]. Among these advantages reduced costs, increased sensitivity and detection range as well as the absence of cross-hybridization are the most prevalent. Using RNA-Seq, transcript abundances can be determined with high accuracy over several orders of magnitude based essentially on the number of reads covering a particular cDNA. However, extensive PCR steps during sample preparation and sequencing may partially bias the obtained results. This is particularly true for transcripts containing nucleotide repeats or GC-rich regions that interfere with polymerase activity [29].

RNA-Seq has been successfully applied to improve genome annotations of several species [15, 30], though transcriptomics of sample material comprising the transcriptome of host and pathogen is still a challenge with respect to correct mapping of reads and the significant disproportion in the amount of reads derived from host and pathogen. The latter applies especially to fungal pathogens with a hemibiotrophic lifestyle which is featured by little or no increase of fungal biomass during the early stages of infection. Despite the excision of inoculated leaf areas as a method to increase the percentage of fungal transcripts, we observed approx. 100-fold more plant than fungal transcripts until 48 hpi. In contrast, later time points showed a significantly improved ratio due to progressive colonization of leaf tissue and spread of infection during necrotrophic development of *C. graminicola* [6]. The relative low number of about 51 million reads that mapped to the fungal genome compared to the overall number of reads (1,310 million) clearly reflects this relationship. Nevertheless, the excellent coverage of transcriptional active regions of 122-fold as well as the overall distribution of reads across transcripts shows sufficient sequencing depth and consistent 5′ to 3′ coverage of transcripts.

Analysis of the read distribution revealed that 14.8% of the reads mapped to intergenic regions. This percentage is somewhat higher compared to 12.9% in *F. graminearum* [15], 3% in *Homo sapiens* [31] and 5% in *Arabidopsis thaliana* [32]. Zhao and associates [15] suggested that a high percentage of intergenic RNA-Seq reads may at least partly reflect the lower quality of gene model predictions in organisms that have either been sequenced recently or their genomes had not been subjected to several rounds of genome annotation as, for example, the well-studied genomes of *H. sapiens* or *A. thaliana*.

About 7% of the RNA-Seq reads could be matched to multiple locations in the genome. Analysis of these reads revealed predominantly rRNA sequences. In addition, 1.6% of all quality-processed reads matched to unique positions in rRNA-encoding sequences indicating the presence of relative high amounts of reads covering rRNA, even after enrichment of mRNAs using oligo(dT) magnetic beads prior to cDNA synthesis. This may be partly due to the fact that hairpin structures in rRNA molecules enabled cDNA synthesis. Another reason could be the presence of poly(A) tails that have been proposed to serve as degradation mark in rRNAs [33, 34].

In contrast, only very few reads that mapped to multiple locations matched coding regions of genes which indicates excellent specificity of our RNA-Seq reads with respect to the corresponding target transcript. Therefore, the numeric read coverage of a particular transcript can be regarded as a reliable reflection of the gene expression level. Consequently, RNA-Seq data collected in the present project can be applied for further studies including analysis of the transcriptional changes during pathogenic development of *C. graminicola* or comparative transcriptomics using different maize cultivars as hosts.

In *C. graminicola*, 14.8% of the RNA-Seq reads fell within intergenic and intronic regions. This relatively large number can be explained by the presence of as yet unrecognized gene transcripts or UTRs and non-coding RNAs. Alternatively, these transcripts correspond to novel exons of known genes which had not been identified as integral part of the ORF.

Among the 819 novel TARs, 106 were likely representing protein-coding genes as evidenced by the presence of full-length ORFs, i. e. there was no stop codon present in the ORF that yielded the blast result. Among the remaining nTARs, only 5 could be identified as transfer, splicesosomal, small nuclear and ribosomal RNAs leaving 82% of the nTARs without a clear function. A possible explanation for this discrepancy is that transcripts without a proposed function could be either species-specific or the corresponding transcripts have not been annotated yet in related species. As a consequence, the role of the vast majority of nTARs remains elusive even though many of those represent most likely non-coding RNAs with regulatory function, pseudogenes or by-products [35, 36].

In addition to several hundreds of nTARs we identified novel candidate exons in 111 annotated genes and revised intronic sequences in 479 genes. Altogether, 906 incorrect gene models were improved based on our RNA-Seq data, which defines a fraction of 7.3% of incorrect gene models deposited in the published annotation of *C. graminicola*. This relatively high percentage of incorrectly annotated genes convincingly shows both the need for manual revision of genome annotations that rely solely on software-assisted prediction of gene models as well as the potential of RNA-Seq as a powerful tool for significantly improved genome annotations.

RNA-Seq data of this study were generated from fungal infection structures sampled at six time points of the infection cycle. This experimental approach was chosen not only to increase the diversity of fungal transcripts but also to enable the detection of infection stage-specific AS. Evidence for AS has been found in many organisms including *H. sapiens, Caenorhabditis elegans*, *Arabidopsis thaliana*, *F. graminearum* and *Aspergillus oryzae*, however, experimental proof that includes sequence data of proteins is scarce for fungal organisms. Whereas about 95% of all genes in *H. sapiens* undergo alternative splicing [37], the fraction of alternatively spliced genes is 42% in *A. thaliana*, 8.6% in A. oryzae, 1.7% in *F. graminearum* and 2.7% in *M. oryzae* [32, 16, 15, 38]. In general, AS events in fungi appear to be restricted to relative few genes compared with higher organisms even though a percentage of 0.6% alternatively spliced genes in *C. graminicola* and almost 9% in *A. oryzae* indicate a surprising variability in related species. This significant spread may at least partially reflect the dilemma of AS analysis. On the one hand, prediction of AS based on transcript sequence data seems to be overestimated in several filamentous fungi as cDNA-derived sequence data without supporting protein sequence data do not allow discrimination between AS and unprocessed or partially processed transcripts. On the other hand, the frequency of AS tends to be underestimated in some studies due to inappropriate experimental conditions that do not reflect the challenges of the natural habitat.

AS has been evolved as a sophisticated mechanism that increases the flexibility of gene expression in order to enable organisms to adapt to various environmental conditions. Experimental approaches dedicated to the analysis of AS should therefore reflect this dependency as the percentage of AS events will certainly increase under more different growth conditions or stresses. To meet these requirements, we collected sample material during pathogenic development of *C. graminicola*. However, only 75 putative AS events in 69 genes could be detected, most of which reduced the size of the corresponding gene product significantly. Although we applied strict criteria in defining putative AS events with respect to the proportion of alternate transcripts, it cannot be excluded that at least some of these candidates constitute unprocessed transcripts rather than isoforms. Moreover, there was no evidence for developmentally-regulated AS thus questioning the relevance of AS in pathogenic development of *C. graminicola*.

## Conclusions

We have analyzed the transcriptome of the corn anthracnose fungus *C. graminicola* during infection of maize leaves by RNA-Seq. Combined trancriptomes of host and pathogen require increased sequencing depth compared to similar approaches with axenic fungal cultures, however, samples of infection-related RNAs display a greater variety of transcripts, which is a precondition for the identification of nTARs and the improvement of hitherto annotated gene models. We obtained an extensive set of RNA-Seq data covering about 98% of the predicted genes which enabled both the revision of incorrect gene models and the identification of hundreds of nTARs. In addition, we obtained preliminary information on alternative splicing in *C. graminicola* thereby questioning the frequency and relevance of AS in filamentous fungi.

Unbiased genome annotations are an indispensable prerequisite for modern research approaches. Combining automated gene annotations and manual revisions of gene models based on sequence data from genomic DNA and mRNA, which has been shown to increase the quality of genome annotations significantly, should therefore become a standard procedure in genome research.

## Methods

### *Colletotrichum graminicola*strain, culture conditions and maize infection

The wildtype strain CgM2 of *C. graminicola* (Cesati) Wilson [teleomorph *Glomerella graminicola* (Politis)] was provided by R. L. Nicholson, Purdue University, IN, USA. CgM2 was cultured on oat-meal-agar plates at 23°C under near UV light (Philips TLD36W/08, Hamburg, Germany).

Conidia were harvested from 2-week-old plates in distilled water and washed three times. The final concentration of conidia was adjusted to 10^6^/ml in 0.01% Tween 20 prior to inoculation of maize plants or leaf segments.

For RNA-Seq, maize plants (*Zea maize* cv. Golden Jubilee) were grown in individual pots for 14 days at 23°C. The 3^rd^ leaf was carefully fixated in a horizontal position and inoculated with 10-μl droplets containing 10^4^ conidia in 0.01% (v/v) Tween20. After 24 h in a moisture chamber at 25°C, plants were transferred to an environmentally-controlled greenhouse cabinet and further incubated at 25°C. Samples corresponding to three biological replicates were taken at 0, 12, 24, 48, 72 and 120 hours postinoculation using a cork borer (8 mm diameter) and immediately frozen in liquid nitrogen. The infection progress was observed microscopically and anthracnose lesions were photographed at 5 days postinoculation.

### Preparation of RNA and Illumina sequencing

Frozen leaf discs (500 mg per sample) were homogenized in 2 ml reaction tubes using pre-cooled steel beads (3 mm) and a tissue lyser (Qiagen, Hilden, Germany) at 30 Hz/60 s. Four hundred and fifty μl buffer RLT (Qiagen, Hilden, Germany) was added to the frozen powder. RNA extraction was performed using the Plant RNA Kit (Peqlab, Erlangen, Germany) according to the manufacturer’s instructions. Quality and quantity of the extracted RNA was examined using Nanodrop 1000 photometer (Peqlab, Erlangen, Germany) and Bioanalyzer 2100 (Agilent, Böblingen, Germany). RNA samples were further processed and subjected to high-throughput sequencing by Illumina HiSeq^TN^2000 at ServiceXS (Leiden, Netherlands).

### Processing of RNA-Seq reads and mapping

The *C. graminicola* CgM2 genome and gene information (version 11/2012, bioproject PRJNA37879) were downloaded from http://www.broadinstitute.org/annotation/genome/colletotrichum_group/MultiDownloads.htm. Reads containing sequencing adapters and reads of low quality were identified by fastqc (http://www.bioinformatics.bbsrc.ac.uk/projects/fastqc). Quality-processing of raw reads included Phred score-guided trimming (Phred score >20) of reads using the software fastx_trimmer of the FASTX toolkit (http://hannonlab.cshl.edu/fastx_toolkit). Elimination of adapter sequences was achieved by removing 12 nucleotides of the 5′-ends of all reads using TrimGalore (http://www.bioinformatics.babraham.ac.uk/projects/trim_galore). All further analysis was done with quality-processed reads.

RNA-Seq reads were mapped against the genome of *C. graminicola* with Tophat version 2.0.8 [39] using default options except for the minimum intron length which was set to 30 nucleotides. Each time point and biological replicate of the maize infection approach was mapped independently, and individual datasets were subsequently joined prior to further analysis.

In order to evaluate the reproducibility of RNA-Seq data obtained from biological repeats we compared the read number per gene of samples belonging to three independent infection series (0-120hpi) using SAMtools [40], htseq-count [41] and R [42].

The average coverage of all cds by RNA-Seq reads was determined using RSeQC [43]. Genomic features were analysed using featureCounts [44] of the software package Subread version 1.4.5. Coverage and distribution of transcriptional active regions was analysed using BEDTools [45] together with custom bash scripts.

A comprehensive scheme illustrating sample preparation and workflow of the *in silico* analysis is provided in the supplement (Additional file 8).

### Transcript assembly and identification of incorrect gene models

Initial transcript assembling was done as described [46]. Additional transcript assembling and gene prediction using introns as hints was performed by AUGUSTUS [20]. Predicted gene models were manually inspected using the Integrative Genomics Viewer (IGV) [19] that enabled simultaneous display of gene models predicted by AUGUSTUS, Cufflinks and Broad Institute together with RNA-Seq reads. For each gene model it was decided whether one of the above annotations was supported by RNA-Seq reads. A manual curation of gene models was performed if none of the gene models was in agreement with our RNA-Seq data.

### Identification of novel transcriptionally active regions

Novel transcriptionally active regions (nTARs) within intergenic regions of the current genome annotation were detected essentially as described in Trapnell and associates [46]. Briefly, nTARs were identified by comparing the current broad annotation with the annotation obtained from the cufflinks pipeline (filtering class code u) using cuffcompare (option -r).

Further characterization of nTARs was performed by blastx search against the non-redundant protein database hosted by NCBI. Subjects with an E-value <10e^-8^ were considered to be putative orthologs.

Nucleotide sequences were translated using TransDecoder (http://transdecoder.sourceforge.net) and analyzed with respect to the occurrence of functional protein domains in the Pfam-A database [48] using HMMER 3.1b1 (threshold parameter -cut_ga) [47], defining an E-value <10e^-3^ as significant hit.

For further characterization, databases fRNA [49] and NONCODE v3.0 [50] both harboring collections of non-coding RNAs were searched using nTAR sequences as input.

### Analysis of alternative splicing (AS)

The improved genome annotation was used to screen for AS in *C. graminicola*. Significant coverage of intron sequences by RNA-Seq reads or locally decreased read numbers in exons were considered to be indicative of AS. Alternative splice sites were required to be supported by a read number of at least 25% uniquely mapped reads compared to the read number of the surrounding exons. Putative AS events were grouped into 5′- or 3′- AS, intron retention and exon skipping. Transcripts displaying insufficient read coverage (read number <30) at junction sites and putative AS events that would lead to very short proteins due to the occurrence of an early stop codon in the alternate ORF were excluded from further analysis.

### Annotation of UTRs

UTRs were annotated based on the predicted gene models of the current genome annotation and RNA-Seq sequence information using BEDTools [45]. To improve signal-to- noise ratio the number of aligned reads was doubled. Positions covered by 0, less than 8 and less than 15 reads had been removed in three independent calculations. The resulting fragments were annotated on the basis of the current genome annotation and fragments containing sequences of adjacent annotated genes were removed. UTRs were considered to be correct if two out of three calculated UTR models resulted in a similar UTR length.

### Screening for RNA editing

Comparative analysis of the genomic sequence of the reference strain CgM2 of *C. graminicola* and our RNA-Seq data was performed in order to identify nucleotide modifications based on RNA editing. Read duplicates in the RNA-Seq data set and in the genomic sequences were removed using PicardTools (picard.sourceforge.net). Nucleotide substitutions and insertions/deletions (INDELS) were detected using SAMTools [40] and snpEff [51]. Candidate sites were required to be covered by more than 30 reads with at least 25% of reads possessing the alternate allele. Each genomic position harboring a putative nucleotide substitution or INDEL was carefully examined with respect to sequence errors or misalignments. Insertions or deletions of one nucleotide in oligo-nucleotide repeats in RNA-Seq data were considered as amplification bias and excluded from further analysis. Differences in the nucleotide sequence between RNA-Seq data and genomic sequence data that could not be explained by misalignments were further analyzed by re-sequencing the corresponding genomic DNA.

### Data deposition

Illumina sequencing data were deposited in the sequence read archive at NCBI (http://www.ncbi.nlm.nih.gov/sra) with accession number SRX516617. The annotation file can be downloaded at http://www.landw.uni-halle.de/download/.

## Electronic supplementary material

Additional file 1: **nTARs. 819 nTARs were identified in intergenic regions.** (XLSX 226 KB)

Additional file 2: **nTARs with similarity to proteins in filamentous fungi.** 146 nTARs were similar to proteins from filamentous fungi. 31 of these nTARs harbored known protein domains. (XLSX 71 KB)

Additional file 3: **nTARs with similarity to non-coding RNAs.** 5 nTARs with significant similarity to non-coding RNAs were identified. (XLSX 44 KB)

Additional file 4: **Incorrectly annotated genes that have been revised.** Gene models of 906 genes that were incorrectly annotated in the Broad *C. graminicola* database have been improved. (XLSX 137 KB)

Additional file 5: **Single nucleotide substitutions, deletions or insertions that do not affect the integrity of annotated genes.** Among the 81 nucleotide substitutions and insertions/deletions identified, 40 had no effects on the integrity of annotated genes due to their localization upstream or downstream of coding sequences (cds) or within nTARs. Silent nucleotide substitutions did not alter the protein sequence due to the degeneration of the triplet code. (PDF 206 KB)

Additional file 6: **Genes with alternative splicing.** 75 putative alternative splice events were detected in 69 candidate genes. (XLSX 38 KB)

Additional file 7: **Identification of UTRs.** 5′- and 3′-UTRs of 4378 genes have been identified. (XLSX 382 KB)

Additional file 8: **Work flow illustrating sample generation, sequencing and analysis of RNA-Seq data.** (PDF 790 KB)

## Abbreviations

nTAR: Novel transcriptionally active region
UTR: Untranslated region
AS: Alternative splicing
INDELS: Insertions/deletions
SNP: Single nucleotide polymorphism.
