# Supplementary figures and images for: New gene models and alternative splicing in the maize pathogen Colletotrichum graminicola revealed by RNA-Seq analysis
Source: BMC Genomics. 2014 Oct 2;15(1):842. doi: 10.1186/1471-2164-15-842 (PMC4194422; doi:10.1186/1471-2164-15-842)

# Additional file 7

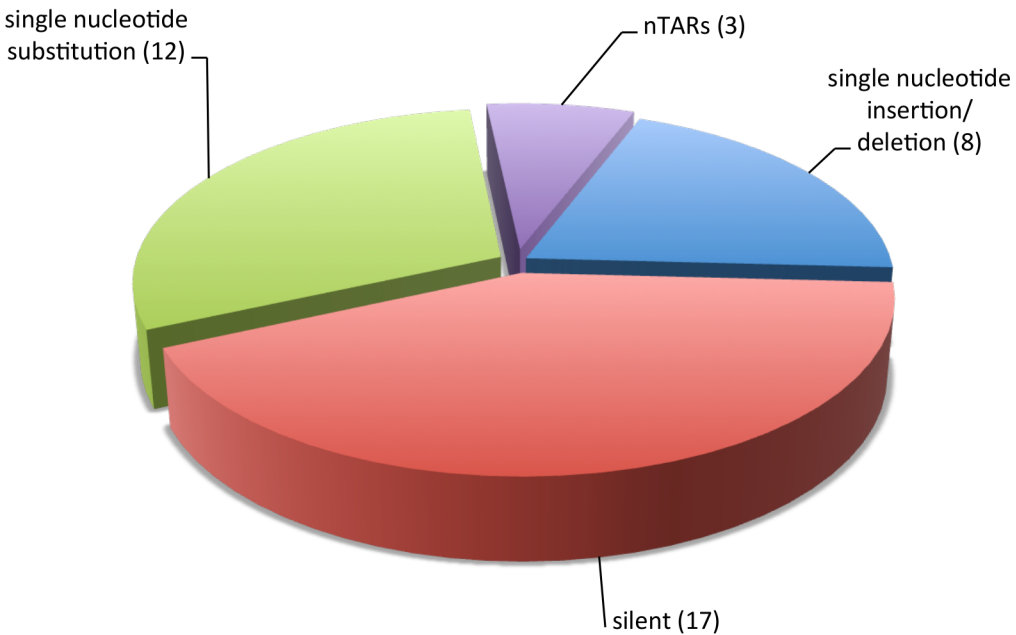

Supplement: Supplementary file 5 — Additional file 5: Single nucleotide substitutions, deletions or insertions that do not affect the integrity of annotated genes. Among the 81 nucleotide substitutions and insertions/deletions identified, 40 had no effects on the integrity of annotated genes due to their localization upstream or downstream of coding sequences (cds) or within nTARs. Silent nucleotide substitutions did not alter the protein sequence due to the degeneration of the triplet code. (PDF 206 KB) [file 12864_2014_6520_MOESM5_ESM.pdf]

# Additional file 8

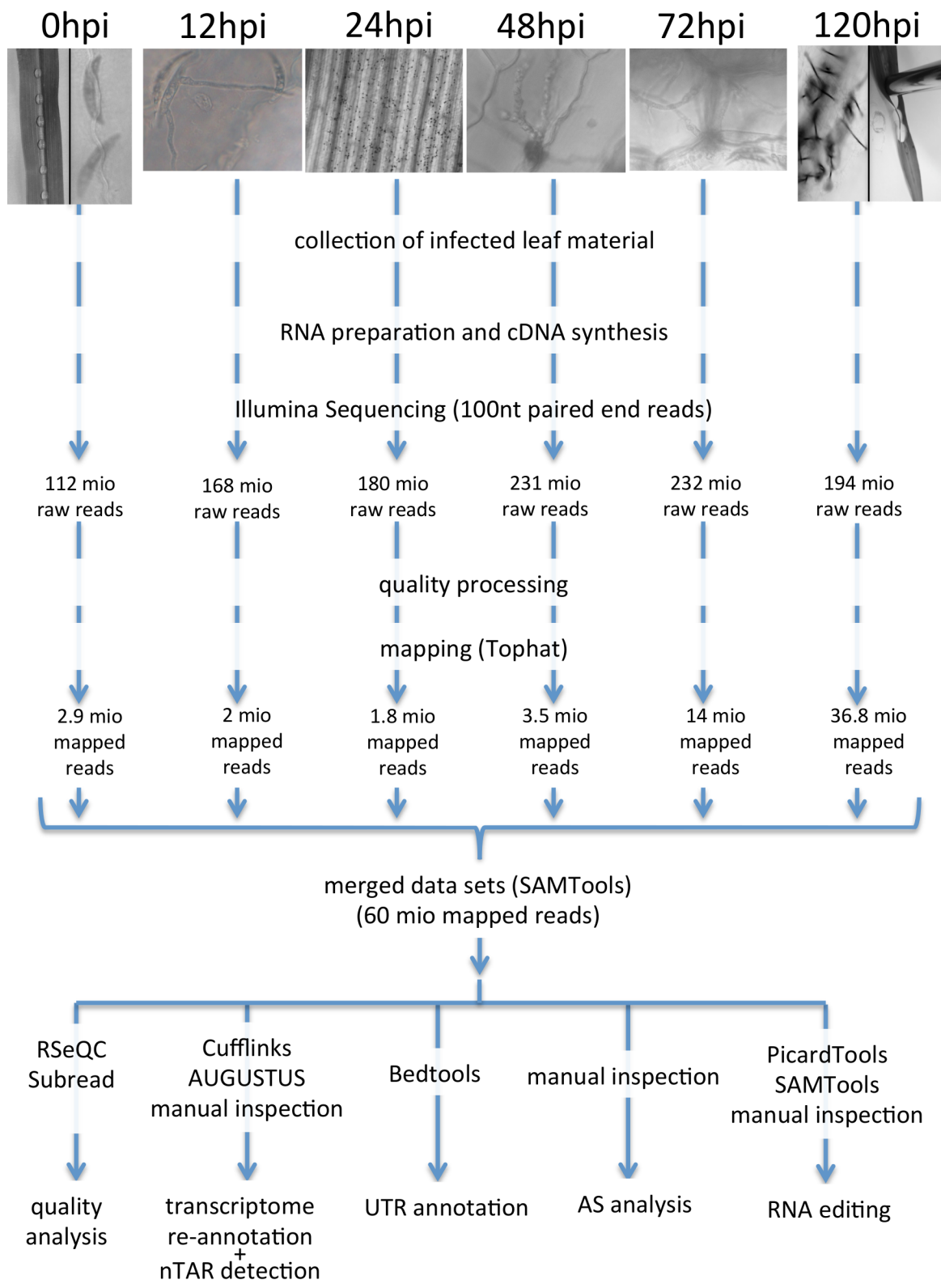

Supplement: Supplementary file 8 — Additional file 8: Work flow illustrating sample generation, sequencing and analysis of RNA-Seq data. (PDF 790 KB) [file 12864_2014_6520_MOESM8_ESM.pdf]
